# Defense Mechanisms against Viral Infection in *Drosophila*: RNAi and Non-RNAi

**DOI:** 10.3390/v10050230

**Published:** 2018-05-01

**Authors:** Luc Swevers, Jisheng Liu, Guy Smagghe

**Affiliations:** 1 Institute of Biosciences & Applications, NCSR “Demokritos”, 15341 Athens, Greece; swevers@bio.demokritos.gr; 2 School of Life Sciences, Guangzhou University, 510006 Guangzhou, China; jisheng.liu@gzhu.edu.cn; 3 Department of Plants and Crops, Faculty of Bioscience Engineering, Ghent University, 9000 Ghent, Belgium

**Keywords:** insect, RNAi, non-RNAi, defense systems, antiviral, insect pest control

## Abstract

RNAi is considered a major antiviral defense mechanism in insects, but its relative importance as compared to other antiviral pathways has not been evaluated comprehensively. Here, it is attempted to give an overview of the antiviral defense mechanisms in *Drosophila* that involve both RNAi and non-RNAi. While RNAi is considered important in most viral infections, many other pathways can exist that confer antiviral resistance. It is noted that very few direct recognition mechanisms of virus infections have been identified in *Drosophila* and that the activation of immune pathways may be accomplished indirectly through cell damage incurred by viral replication. In several cases, protection against viral infection can be obtained in RNAi mutants by non-RNAi mechanisms, confirming the variability of the RNAi defense mechanism according to the type of infection and the physiological status of the host. This analysis is aimed at more systematically investigating the relative contribution of RNAi in the antiviral response and more specifically, to ask whether RNAi efficiency is affected when other defense mechanisms predominate. While *Drosophila* can function as a useful model, this issue may be more critical for economically important insects that are either controlled (agricultural pests and vectors of diseases) or protected from parasite infection (beneficial insects as bees) by RNAi products.

## 1. Introduction

RNA interference (RNAi) is considered an ancient gene silencing pathway linked to antiviral defense [1]. Small RNA-guided antiviral immunity was first demonstrated in plants [2] and subsequently in *Drosophila* [3] and *Caenorhabditis elegans* [4].

In *Drosophila*, the major RNAi pathway involved in antiviral immunity is initiated by the processing of virus-derived dsRNA molecules to viral small interfering RNAs (viral siRNAs or vsiRNAs) by Dicer-2 (Dcr-2) enzyme. Viral siRNAs are subsequently loaded in an effector complex named RISC (RNAi-induced silencing complex) with Argonaute-2 (Ago-2) as central molecule. SiRNA-programmed RISC complexes subsequently scan cellular RNA populations for complementary sequences and cause specific RNA degradation after specific siRNA-mRNA hybridization [5]. The central factors of the siRNA pathway, Dcr-2 and Ago-2, were demonstrated to have undergone accelerated evolution as a consequence of adaptive virus-host arms races [6]. The other RNAi pathways in insects, characterized by microRNAs (miRNAs) and Piwi-associated RNAs (piRNAs), have recently also been shown to be involved in antiviral defense [7]. However, the siRNA pathway is considered the major antiviral RNAi pathway in insects [8].

While the piRNA pathway is restricted to germline tissues [9,10], in somatic tissues the miRNA (characterized by Dcr-1/Ago-1) and siRNA pathways (characterized by Dcr-2/Ago-2) are maintained independently. In contrast to miRNA-dependent Ago-1-RISC, the efficient assembly of siRNA-dependent Ago-2-RISC requires the RISC-loading complex, consisting of Dcr-2, the dsRNA-binding protein R2D2 and TATA-binding protein-associated factor 11 (TAF11), an unannotated basal transcription factor [11]. Reconstitution of Ago-2-RISC assembly in vitro further shows the requirement of the chaperone machinery (Hsc70-4, Hsp83, Hop, Droj2, p23; dependent on ATP) which is viewed to occur in analogous fashion to steroid hormone receptor maturation (and with siRNA duplexes as ligands) [12]. Maturation of the pre-RISC complex or RISC activation occurs after cleavage of one of the strands of the siRNA duplex by slicer activity of Ago-2 and the endonuclease C3PO [13]. Separation of miRNA and siRNA pathways is further evident by the localization of their components in different subcellular membrane-less organelles (P-bodies or GW-bodies for Ago-1-RISC [14]; D2-bodies for Ago-2-RISC [15]). The separation of the miRNA from the siRNA machinery in the cellular cytoplasm may be driven by the necessity to avoid interference in the managing of disparate RNAi functions (maintenance of cellular gene networks versus innate immunity).

Homozygous mutants for *dcr-2* and *ago-2* are viable and fertile, indicating that these core siRNA components are not required for viability and development [16,17]. Mutants for *r2d2* that survive to adulthood also show normal morphology and behavior but were found to have reduced female fertility by a mechanism that does not involve its function in the siRNA pathway [18]. On the other hand, over-expression of Dcr-2 was reported to increase gene silencing by RNA hairpins in transgenic flies [19]. Other studies implicate a link between nutrient conditions and robustness of the RNAi response. When energy levels are low and insulin/insulin-like growth factor signaling is reduced, the forkhead transcription factor dFOXO responds by translocation to the nucleus resulting in increased target gene expression [20]. It was observed that the induction of dFOXO in transgenic flies results in increased expression of the RNAi machinery genes *ago-1*, *ago-2* and *dcr-2* and concomitant resistance to virus infection [21]. In dFOXO null flies the greater susceptibility to RNA viruses can be rescued by over-expression of Dcr-2. The increase in RNAi efficiency in cultured cells after serum starvation may occur through a similar mechanism [22]. These data indicate that the efficiency of RNAi-mediated silencing is not constant and linked to cellular physiology and homeostasis.

Besides RNAi, many other innate immune pathways have been proposed to be involved in antiviral defense such as the Toll and Imd pathways, originally identified for their involvement in antibacterial and antifungal defense, the JAK/STAT pathway, translational inhibition, transcriptional pausing, autophagy, heat-shock response, apoptosis, phagocytosis of infected cells and phenoloxidase activity (see references [23,24,25,26,27,28,29,30,31] for examples from different insect species) [23,24,25,26,27,28,29,30,31]. Sloughing off infected gut cells has also been reported to clear infections of baculovirus [32]. Very recently, a detailed review was also published that discusses the sources of variation in resistance to virus infection in dipteran insects [33]. An interesting question relates to the relative importance of each of the proposed innate immune response pathways to control viral infections. Research to answer this question has already revealed that the specific antiviral response is both insect host- and virus-dependent [34,35].

Control mechanisms may differ between pathogenic and persistent infections. Virulent pathogenic infections may be initially controlled by the host but ultimately will prevail as the virus provides a powerful machinery for viral replication and innate immune suppression. During persistent infections, on the other hand, a state of equilibrium seems to be established between viral maintenance and immune surveillance. Persistent infections present interesting cases because of their long-term interactions with the host, which could change its physiology, including immune pathways such as RNAi.

The relative importance of the RNAi pathway to control viral infections may be relevant for the use of RNAi to achieve gene silencing in reverse genetics experiments or in the application of RNAi for pest control. It can be assumed that viruses may evade different types of immune response in a differential manner, with some viruses evading primarily RNAi and some viruses mainly other defense pathways. If a virus escapes control by the RNAi pathway, other defense pathways will evolve to control the virus, which could lead to a temporal decrease in the efficiency of the antiviral RNAi machinery. An interesting research avenue would be to investigate whether the relative importance of RNAi to control viral infections may indicate its relative robustness to support endogenous gene silencing efforts. Here, we review the variability of the immune response against viral infections in the model insect *Drosophila melanogaster*, in order to show that many other antiviral strategies can exist besides RNAi. When a multitude of responses exist, it is possible that particular non-RNAi responses can dominate at the expense of the contribution of RNAi. Such evolution of antiviral immune response mechanisms, which so far have not been investigated directly, may have implications for practical applications of RNAi in insects, such as RNAi-based gene silencing experiments, control of pest insects in agriculture and medicine, and increasing the health of beneficial insects such as bees.

## 2. Paradigm: RNAi as Antiviral Defense Mechanism in Drosophila

### 2.1. RNA Viruses

Because of its extensive genetic resources, the fruitfly *D. melanogaster* was used as a model to investigate the involvement of the RNAi pathway in antiviral defense. Three major criteria are applied to indicate the interaction between virus infection and RNAi: (1) the production of viral siRNAs (vsiRNAs; typically, 21 nt in *Drosophila*) characteristic of processing by Dicer; (2) increased viral replication and mortality in *dcr-2* and *ago-2* mutants; (3) the existence of viral suppressors of RNAi (VSRs) in viral genomes (reviews by [36,37]).

The production of abundant 21 nt vsiRNAs during infection has been documented in *Drosophila* tissues as well as the *Drosophila*-derived Schneider-2 (S2) cell line for many viruses with a positive strand ssRNA genome (e.g., Flock house virus (FHV) and other nodaviruses, the dicistroviruses Drosophila C virus (DCV) and Cricket paralysis virus (CrPV), Nora virus (Picornavirales)) and several viruses with a dsRNA genome (e.g., Drosophila X virus (DXV, *Birnaviridae*)) (reviews by [7,8,27,36]). In S2 cells, approximately equal numbers of vsiRNAs were derived from genomic and antigenomic strands, implicating an origin from replication intermediates or complete genomes in the case of dsRNA viruses [38]. The vsiRNAs that are produced are functional since they inhibit reporter gene activity in appropriately designed sensor assays (constructs that connect the luciferase ORF with sequences of the viral genome [39]). Interestingly, even when vsiRNAs accumulate at much lower levels, such as in *loqs* mutants, viral infection can be controlled efficiently, indicating the efficiency of RNAi antiviral immunity can be relatively insensitive to the abundance of vsiRNAs [40]. As expected, flies that were mutant for *dcr-2*, *ago-2* or *r2d2* were more susceptible to RNA virus infection, manifested by increased mortality and higher virus titers [5,34,41].

In all the above-mentioned RNA viruses (FHV, DCV, CrPV, Nora virus, DXV), VSRs were identified, that can block the RNAi mechanism either at sensor or effector levels. The B2 protein of FHV binds both dsRNA and siRNA with high affinity, protecting it from Dicer activity [42]; in addition, the C-terminus of B2 can interact with Dicer-2 to prevent the loading of RISC with siRNA [43,44]. For dicistroviruses, the N-terminal 99 amino-acids of DCV (DCV-1A protein) acts as a VSR through binding of dsRNA and to a lesser extent siRNA by its dsRNA-binding motif, while the N-terminal CrPV-1A protein (148 amino-acids) binds Ago-2 and interferes with the function of holo-RISC [5]. Similar to CrPV-1A, the VSR of Nora virus, VP1, inhibited slicer activity of pre-loaded RISC in cellular and embryonal extracts [45]. Interestingly, Nora virus of *Drosophila immigrans* was only capable to inhibit slicer activity in *D. immigrans* extracts and not in *D. melanogaster* extracts and to interact with conspecific Ago-2, indicating host-specific evolution of the VSR protein [39]. Finally, the VP3 protein of entomobirnaviruses such as DXV and Culex Y virus, characterized by two linear dsRNA genome fragments, represents a multifunctional protein that also interacts with dsRNA to form ribonucleoprotein complexes, as such simultaneously acting as a VSR [46,47].

It was also observed that RNA virus infection or ectopic expression of VSRs can inhibit dsRNA-mediated silencing of cellular or reporter genes (e.g., DCV and CrPV [5,41]; DXV [47]), although this may depend on the viral infection levels and achieved VSR expression levels. VSR activity has also been demonstrated to interfere with the endo-siRNA pathway (which controls expression of transposable elements [5]), while the miRNA pathway remains largely unaffected during viral infection in insects [5,41,45]. The latter situation contrasts with RNA virus infection in plants where interference with miRNAs can lead to physiological and developmental defects, which contribute to viral disease [48,49].

### 2.2. Arbovirus Infections in Drosophila

Flies and *Drosophila*-derived cell lines have been extensively used as models to study infections of arboviruses that naturally are vectored by mosquitoes [29]. Those studies do not only include infections with RNA viruses with positive-strand ssRNA genome (e.g., Sindbis virus and Semliki Forest virus (SINV and SFV; both *Alphavirus*, *Togaviridae*)), but also with negative-strand ssRNA genome (e.g., Vesicular stomatitis virus (VSV; *Rhabdoviridae*)) and segmented negative-strand/ambisense ssRNA genome (e.g., Rift Valley Fever virus (RVFV; *Bunyaviridae*)) (reviews by [7,8,27,36].

While arbovirus infections can cause severe disease in mammalians, arboviruses cause no or only mild pathogenic effects in mosquito vectors. Such non-pathogenic, persistent state of infection by arboviruses occurs also in mosquito- and *Drosophila*-derived cell lines [50]. As for the other RNA viruses, infections with arboviruses also result in the production of 21 nt vsiRNAs, indicative of processing by Dicer-2. For alphaviruses (SINV and SFV), vsiRNAs were equally distributed between genomic and antigenomic strands and therefore likely originate from replication intermediates with dsRNA structure [51,52]. Also, VSV infections produce 21 nt vsiRNAs that are approximately evenly distributed between genomic and antigenomic strand [51,53]. Sensor assays established that vsiRNAs from VSV infections can efficiently knock down engineered reporter constructs, indicating their loading in functional RISC complexes [53]. On the other hand, it was also observed that defective interfering (DI) particles can be produced during VSV infections. DI particles correspond to a 1.6 kb region at the 5′-end of the VSV genome and are the source of abundant vsiRNAs [51]. Interestingly, knockdown of *ago-2* results in a decrease in vsiRNAs outside the 1.6 kb region, while an increase is observed within the 1.6 kb region. Because Ago-2 stabilizes siRNAs, this result is interpreted that the abundant vsiRNAs, likely derived from DI particles, are not loaded in Ago-2 and therefore non-functional [54]. Finally, RVFV 21 nt vsiRNAs, isolated after infection of *Drosophila* cells, distribute more or less equally between positive and negative RNA strands for the M and L segments. In the S segment, a large fraction of the vsiRNAs map to a particular region in the antigenome that resembles a stem-loop [54].

*Drosophila* flies mutant for *dcr-2* and *r2d2* (but not *loqs*, involved in the miRNA pathway) showed increased levels of SINV genomes and for *r2d2* mutants also a decrease in lifespan was observed [51]. VSV infections also resulted in increased mortality in *dcr-2*, *r2d2* and *ago-2* mutant flies that could be correlated with a large increase in viral titers [53,54].

Given their propensity to establish persistent infections, it is under debate whether arboviruses encode VSR proteins. For instance, when the alphavirus SINV is engineered to express a heterologous VSR, such as VP1 from Nora virus, increased mortality is observed after injection in wild-type flies [45]. By contrast, no differences in mortality are observed between control SINV and SINV-VP1 in *dcr-2* mutants, indicating that the increased pathogenicity of SINV-VP1 results from suppression of the RNAi mechanism. In the case of SINV, the absence of a VSR gene in its genome therefore clearly correlates with its propensity to establish persistent infections that do not cause pathogenic effects. However, for other arboviruses, such as flaviviruses, strong evidence exists that they encode a VSR protein. Using the same experimental approach as discussed above, it was demonstrated that the capsid protein of West Nile virus and other flaviviruses could function as a VSR in the context of infections of mosquitoes with recombinant SINV [55].

### 2.3. DNA Viruses

Invertebrate iridescent virus 6 (IIV-6; *Iridoviridae*), a complex virus with a large dsDNA genome of >200 kb encompassing >200 ORFs, was used as a model for DNA virus infections in *Drosophila* [36,56]. While the virus is not specific to *Drosophila*, it has a broad host range and can cause pathogenic infections in flies.

During infection of flies with IIV-6, 21 nt vsiRNAs are produced that are produced from hot spots on the viral genome [56]. Further analysis indicated that vsiRNAs are derived from overlapping sense and antisense transcripts (forming dsRNA structures) rather than from strong stem-loop structures in single-stranded mRNAs. Sensor assays indicated that the vsiRNAs produced during IIV-6 infection were fully functional for RNAi.

IIV-6 infection of *dcr-2*, *r2d2* or *ago-2* mutant flies resulted in higher mortality [34], which, however, was accompanied with only a modest increase in viral titers [56]. Furthermore, ORF340R protein, which contains a canonical dsRNA-binding domain (dsRBD), was identified as a VSR [36]. ORF340R binds both dsRNA and siRNA and therefore impairs both processing by Dicer-2 and RISC assembly. VSR activity is rather strong since dsRNA-mediated silencing of a reporter gene is inhibited during IIV-6 infections [36]. On the other hand, the absence of a 22 nt peak during infections of *dcr-2* mutant flies suggests that no viral miRNAs are produced by IIV-6 [56].

Vaccinia virus (VACV) is a mammalian cytoplasmic DNA virus (*Poxviridae*) that also has a complex dsDNA genome of 200 kb. During infection of *Drosophila* cells, it cannot undergo a complete replication cycle; nevertheless 21 nt vsiRNAs are produced that are dependent on Dicer-2 [54]. The genomic termini of VACV that contain 30 tandem repeats of a 70 nt element with hairpin structure, were identified as the source of the most abundant vsiRNAs. Interestingly, VACV infection of *Drosophila* cells also leads to addition of non-templated adenosines at the 3′-end of (Ago-1-loaded) miRNAs that causes their degradation [57]. This phenomenon was also observed during infection of lepidopteran cells derived from the tiger moth *Amsacta moorei* during entomopoxvirus infection and may have evolved as an additional antiviral mechanism [57].

### 2.4. No Involvement of the piRNA Pathway in Antiviral Defense

In contrast to mosquitoes [58], there is no convincing evidence that the piRNA pathway is involved in antiviral defense in *Drosophila*. In *Drosophila*, expression of the main components of the piRNA pathway is restricted to the gonads, where it is involved in the silencing of transposable elements [9,10]. Ovarian tissue can be divided in two cell types, germline cells (oocyte and nurse cells) and somatic support cells, which differ with respect to the piRNA “module” that is expressed. Somatic support cells and the derived ovary somatic sheet (OSS) cell line, express the “linear” piRNA module, in which primary piRNAs are loaded in nuclear Piwi to mediate transcriptional silencing of transposons. Germline cells, on the other hand, are characterized by an efficient mechanism of posttranscriptional silencing in the cytoplasm through the abundant production of secondary piRNAs by a “ping-pong” amplification mechanism executed by the Piwi-class Argonaute proteins Aubergine (Aub) and Ago-3 [59]. Small RNA deep sequencing established that OSS cells are persistently infected with RNA viruses, including DCV, DXV, Nora virus and American Nodavirus (ANV; closely related to FHV), and that 21 nt vsiRNAs can be readily detected [38]. Interestingly, a second class of 24–30 nt viral small RNAs is also present that exhibits a strong bias both for sense polarity (genomic strand for DCV, Nora virus and ANV, and mRNA for DXV) and for uridine at the 5′-end, a hallmark for piRNAs. The viral piRNAs (vpiRNAs) are produced by the “linear” (primary) piRNA pathway, as expected for the OSS cell line, while no evidence was found for the “ping-pong” amplification mechanism [38].

By contrast, a comprehensive study, which investigated viral infections in piRNA mutant flies (*Piwi*, *Aub*, *Ago-3* and *Zucchini* (*Zuc*)), did not observe any increase in virus accumulation or mortality compared with wild-type flies [60]. No clear evidence for production of vpiRNAs was found, even in *dcr-2* and *ago-2* mutants, thus ruling out a possible compensating role for the piRNA pathway in the absence of the siRNA pathway [60]. No differences were observed between wild-type and piRNA mutant flies during acute or persistent infections, infections with RNA or DNA viruses, or infections with viruses that are naturally vertically transmitted such as Sigma virus (*Rhabdoviridae*), and therefore replicate in the gonads. In the study, care was taken to isogenize all fly piRNA mutant lines before experimental manipulation to minimize effects of different genetic background that could have affected the results of earlier studies, wherein a higher susceptibility of *piwi* or *aub* mutant flies to viral infections were reported [61,62]. The observation of vpiRNAs may, therefore, be a peculiar feature of the OSS cell line that differs from the situation in adult flies.

### 2.5. DNA Viruses and Their Interaction with the miRNA Pathway

Interaction with the miRNA pathway has been mostly documented for large DNA viruses, such as baculovirus [63], while its role during RNA virus infections is less clear [64,65]. Because virus research in *Drosophila* has focused on RNA viruses, reports on interactions with host miRNAs or production of viral miRNAs are scarce. An interaction with the miRNA pathway was described during infections of *Drosophila* cells with the mammalian Vaccinia virus (VACV; *Poxviridae*) [57]. Deep sequencing of viral small RNAs also detected an abundant miRNA that is produced during infection with Kallithea virus, a large DNA virus (*Nudiviridae*) [39]. Extension of research on antiviral immunity to DNA viruses is therefore expected to highlight the importance of the miRNA pathway.

### 2.6. RNAi in Combination with Other Degradation Pathways

Next generation sequencing indicates that virus infections generate unique patterns of viral small RNAs, defined by the abundance of reads for each size between 15 and 35 nt [66,67]. The diversity of viral small RNA patterns is consistent with the diverse origins of small RNAs that are generated by the siRNA pathway (21 nt vsiRNAs), the piRNA pathway (27–28 nt vpiRNAs; not important in *Drosophila* but prominent in mosquitoes) and non-specific RNA degradation pathways (no size enrichment but biased towards the viral genomic strand). Virus-specific small RNA patterns are the result of the interaction between divergent strategies of viral replication with host-specific antiviral responses and have been used for virus classification and identification of new virus species [66]. The possible role of RNA degradation pathways that are different from RNAi will be discussed further below (Section 3.2).

### 2.7. Systemic Antiviral RNAi-Based Immunity

RNAi is considered cell-autonomous in insects, including *Drosophila*, which means that the silencing process is limited to the cells in which the dsRNA is introduced or expressed [68]. In transgenic *Drosophila*, silencing effects that result from RNA hairpin transgenes are strictly restricted to the expressing cells and do not extend to neighboring cells [69]. The absence of robust systemic effects was attributed to the absence of an RNAi amplification mechanism (RNA-dependent RNA polymerase homolog) in insects and a dsRNA transport mechanism (SID-1 dsRNA transporter homolog) in dipterans [70].

However, addition of dsRNA to the cell culture medium or through injection in flies demonstrated systemic silencing effects, indicating the existence of a functional dsRNA uptake pathway [22,71]. In S2 cells, functional RNAi-based screens identified receptor-mediated endocytosis as the major pathway for gene-silencing through “dsRNA soaking”, i.e., in the absence of transfection agent [72,73]. The screens identified genes involved in the endocytotic pathway, the oligomeric Golgi complex, cytoskeleton organization, protein transport, lipid metabolism and modification, and with unknown function [72]. In addition, the scavenger receptors SR-CI and Eater were identified as the membrane proteins to interact with exogenous dsRNA and mediate its internalization [73]. Interestingly, SR-CI and Eater are preferentially expressed in macrophage-like hemocytes, which agrees with the observed more efficient silencing in this cell type following dsRNA injection [71].

These studies were subsequently extended to adult flies where genes involved in dsRNA uptake in S2 cells, such as *egghead*, *CG4572* and *ninaC*, were shown to be required for antiviral defense against DCV and SINV infections [74]. It was therefore hypothesized that dsRNA released by lysed cells during infection could be taken up by other cells and initiate a systemic antiviral response in the whole organism [75]

The latest studies however confirm a pivotal role for hemocytes in RNAi-mediated antiviral defense that is both systemic and adaptive [76] (for a more extensive discussion on the role of hemocytes in non-RNAi aspects of antiviral immunity, see also Section 3.8). Hemocytes are not efficiently infected with SINV, but are proposed to acquire viral dsRNA through phagocytosis of dying cells from other tissues [30] or direct endocytosis from the hemolymph. Uptake of dsRNA in combination with viral infection subsequently initiates an amplification mechanism in the hemocytes that comprises the generation of viral DNA forms (after reverse transcription by endogenous retrotransposons; [77]) that function as templates for the production of secondary viral siRNAs as an amplification mechanism [78]. Secondary viral siRNAs provide systemic protection after their secretion in exosomal-like vesicles that are formed from multivesicular bodies. Exosomes generally are implicated in cell-cell communication and transmission of disease states, i.e., through the transfer of small RNAs [79]. In this model, the production of viral DNA forms provides a type of immune “priming” (or memory) that confers protection against future infections of the same (but not other) viruses [76]. In addition, exosomal-like vesicles can provide passive immunity since their transfer from infected flies will protect non-infected flies from viral infection.

The notion of “immune priming” may be a variable process and dependent on the type of virus since it was not observed in other studies [80]. In addition, other mechanisms for a systemic antiviral RNAi-based system were proposed, based on nanotube-like structures [81].

### 2.8. RNAi in Persistent Virus Infections

Viruses are usually associated with causing disease, but deep sequencing efforts have revealed the existence of many “persistent” virus infections in insects, including *Drosophila* [67], that occur without obvious fitness costs to the host. In persistent infections, host and virus use attack and counter-attack until equilibrium is reached where viral replication is controlled but not eliminated [77]. The establishment of persistent infections therefore is dependent on two major factors: (1) repression of viral replication such that pathogenic effects are avoided, and (2) the evasion or suppression of the immune response [82,83,84].

The classic example of persistent infection is the arbovirus infection of mosquitoes, in which viral replication is suppressed by the RNAi machinery to such levels that pathogenicity is avoided [50]. If arboviruses are engineered to express an RNAi inhibitor, persistent infections are transformed into pathogenic infections that cause mortality to the mosquito hosts [45]. Most research on the mechanism of viral persistence in *Drosophila*, however, is based on infections of cell lines with the model virus FHV (*Nodaviridae*) that encodes a well-characterized RNAi inhibitor (Section 2.1; [42,43]. When *Drosophila* culture cells are acutely infected with FHV, extensive lysis and mortality is observed. However, a small proportion of cells survives and establishes a persistent infection that is characterized by low level replication and absence of cytopathic effects [85]. Nevertheless, the virions produced by the cells retain their full infectivity as they were shown to cause mortality in flies with the same rate as virions produced during acute (pathogenic) infections [77]. This observation indicates that the state of persistence occurs at the level of the physiology of the cells rather than through changes in the viruses themselves.

The mechanism of persistence of FHV in *Drosophila* cell lines seems to involve several factors. One mechanism that was proposed most recently was the establishment of an amplification of the RNAi response through the formation of viral DNA forms. In this mechanism, FHV viral RNA is reverse transcribed in conjunction with endogenous retrotransposons to DNA, which is maintained stably in the cells and functions as a continuous source of viral dsRNA/siRNAs that control the infection [77]. The generation of viral DNA forms was also demonstrated for infections of other RNA viruses such as DCV, DXV and SINV.

Staining of viral proteins however showed the different “character” of persistent infection as a different intracellular distribution was observed between persistent and lytic infections [86]. The altered subcellular distribution may represent a suboptimal environment for replication and virion formation during persistence. The distribution pattern of viral siRNAs along the viral genome during persistent infection resembles the pattern observed during acute infections with FHV that has a deletion of the B2 protein [40,87], indicating increased susceptibility to the RNAi machinery.

Another contributing factor may be the production of defective interfering (DI) RNAs that continue replicating in the presence of viral RNA polymerase and preferentially may accumulate during persistent infections. DI RNAs interfere with the infection process through dampening of viral replication and mis-incorporation into viral capsids [86,88]. Furthermore, DI RNAs can be a source of both abundant small RNAs with the potential to target genomic viral RNAs for degradation as well as truncated proteins with inhibitory effects on the infection process.

Besides RNAi, other mechanisms play a role to establish persistent infections. It was shown that activation of the phosphatidylinositol-3-kinase-Akt pathway, which is associated with growth factor signaling and plays an important role in cell proliferation and survival, can increase infections of SINV [89]. Replication of SINV was dependent on the levels of Akt expressed in the cells and, reciprocally, SINV infection resulted in increased phosphorylation of Akt and its target glycogen synthase kinase β. Through the increased expression of the cap-binding complex, the infected cells can accommodate translation of capped viral mRNAs without significant disruption of cellular function which is essential for the persistence of the infection.

## 3. Innate Antiviral Immunity beyond RNAi

While RNAi is generally recognized as a major antiviral pathway in *Drosophila*, it has equally been realized that many other defense mechanisms exist [27,28]. The question can then be raised regarding the relative importance of RNAi versus other defense pathways. More specifically, it can be asked whether such alternative defense mechanisms could provide protection against viral infection in the absence of the RNAi. In addition, disablement of antiviral RNAi defenses can provide the basis for susceptibility to virus infections, as documented for infections of *C. elegans* by Orsay virus [90]. The possible existence of insects with a deficient antiviral RNAi pathway can also have practical importance from the point of view that RNAi is also developed as a new method for pest control [91,92]. In the next sections, an overview is presented of the “non-RNAi” antiviral defense mechanisms in *Drosophila* and if evidence exists whether viral infections can be controlled in the absence of RNAi.

### 3.1. Mutations in Drosophila Populations that Confer Resistance against Natural Viral Pathogens

Genome-wide association studies have revealed polymorphisms that have major effects on resistance against viruses that naturally infect *Drosophila*, but not against other viruses [93,94]. Through long-term evolution with natural infections, viral resistance can emerge either by changing the immune system (at the level of “antiviral genes”) or by altering host factors that are used by the virus during its replication cycle (at the level of “proviral genes”) [95].

The studies focused on two natural viral pathogens of *D. melanogaster*. *D. melanogaster* Sigma virus (DMelSV; *Rhabdoviridae*) is a host-specific pathogen that is only transmitted vertically through sperm of egg. Infection by DMelSV is considered benign although reduced fitness can be observed. DCV (*Dicistroviridae*), of which the interaction with the RNAi machinery has been investigated extensively (see above), on the other hand, can infect a range of *Drosophila* species through feeding. Oral infection of DCV in adult flies can cause 25% lethality after a period of 20 days [96].

Three major loci are associated with resistance against DMelSV. First, a transposon insertion and further arrangements at *ref(3)D* are associated with increasing levels of resistance against DMelSV [97]. Interestingly, mutations in the locus also increase resistance to organophosphate insecticides [98] and the genes affected (*CHKov1* and *CHKov2*) are characterized by a choline kinase domain, possibly linking the resistance mechanism to the level of viral entry since the acetylcholinesterase receptor can function as a cellular receptor for other rhabdoviruses [99]. The second gene is known as *ref(2)P* or *p62* and encodes an adaptor protein of which one of the functions is the selective targeting of polyubiquitinated protein substrates for degradation by autophagy [93,100]. The involvement of *p62* in DMelSV defense could therefore occur through its role in autophagy, a process that is known to protect against other rhabdovirus infections [101] (see also Section 3.2.2 and Section 3.8). Interestingly, *ref(2)P/p62* also forms a complex with atypical protein kinase, which stimulates the innate immune Toll signaling pathway and the induction of antimicrobial peptides (AMPs) [102,103] (see also Section 3.3). The third gene, *ref(2)M*, encodes Ge-1 that plays a role in processing of RNA and the formation of P-bodies or GW-bodies [93,104]. While Ago-2 is also a component of P-bodies [105] and *ago-2* mutants show increased titers of DMelSV, no genetic interaction was found between *ago-2* and *Ge-1* [93]. On the other hand, Ge-1 is known to interact with Decapping protein 1 (Dcp1), an enzyme that removes the 5′-caps from mRNAs and also localizes to P-bodies. Furthermore, knocking down of Dcp1 also results in higher titers of DMelSV. Thus, the resistance mechanism by Ge-1 is hypothesized to be at the level of degradation of viral genomic RNAs or mRNAs in P-bodies in an RNAi-independent manner ([93]; see also Section 3.2.4).

On the other hand, a single gene, *pastrel*, is the dominant factor that regulates susceptibility to DCV infections [95,106]. In this case, resistance is achieved through higher expression levels of *pastrel*, independent of which allele was used. However, while *pastrel* was reported to participate in protein secretion and also associates with lipid droplets, the molecular mechanism in viral resistance remains unknown.

The identified resistance genes act very specifically against DMelSV and DCV infections. Genes that affect DMelSV resistance have no effect on the infectivity of the closely related *D. affinis* Sigma virus (DAffSV) as well as of DCV, FHV or *Drosophila* A virus (DAV; related to *Permutotetraviridae*); in addition, *pastrel* mutations do not influence FHV, DAffSV and DMelSV infections [95,106].

On the other hand, when a *Drosophila* population was experimentally selected for resistance against DCV infection, the evolution of resistance to DCV also led to partial protection against CrPV and FHV infections [107]. Protection was strong against CrPV (a dicistrovirus closely related to DCV) but only moderate against FHV while no significant increase in resistance was found against bacterial infections. Mapping of the resistance alleles revealed that adaptation to DCV and cross-resistance to other viruses relies on a few major genes. The most significantly differentiated genetic change mapped to the *pastrel* gene (also identified in genome-wide association studies mentioned above) which was associated with increased protection against the dicistroviruses CrPV and DCV but not against the nodavirus FHV [107]. Two other genetic changes were located in the gene *Ubc-E2H* (encoding Ubiquitin-conjugating enzyme E2H) which was also associated with specific protection against dicistrovirus infection. By contrast, RNAi knockdown identified *CG8492* (encoding lysozyme) with decreased survival to DCV and FHV but not to CrPV. It is noted that the variation in virus resistance is based on genes that are unrelated to the canonical antiviral defense pathways such as RNAi.

### 3.2. Identification of Host Factors that Restrict Viral Infection by RNAi Screens in Cultured Cell Lines

Following up on the observation that the addition of long dsRNAs to the medium of *Drosophila* culture cells results in specific gene knockdown, this cell culture system was adapted to perform large scale RNAi screening assays to identify genes involved in particular cellular processes [108,109]. This approach was also used to identify host factors that either facilitate or restrict virus infection (viral sensitivity factors (VSFs) or viral resistance factors (VRFs), respectively [110]). Because of their importance to human health, emphasis was placed on arboviruses that are transmitted by mosquitoes but also can establish persistent infections in *Drosophila* cell lines and adult flies, such as SINV (*Alphaviridae*), RVFV and La Crosse virus (LACV) (*Bunyaviridae*), Dengue virus (DENV) and different strains of West Nile virus (WNV), such as the Kunjin strain (KUN) (*Flaviviridae*) and VSV (*Rhabdoviridae*) [111]. The *Drosophila*-specific pathogen DCV was also often included in the screens because it could serve as a model for pathogenic enterovirus infections in mammals [112], while other *Drosophila*- (Nora virus) or insect-specific viruses (FHV) were rarely included. Besides genome-wide screens, also targeted screens were performed, for instance targeting specific signaling pathways, immune genes, RNA helicases, RNA metabolism and endoplasmic reticulum (ER)-associated proteins [101,113,114,115,116].

Because the RNAi technology has major limitations associated with weak silencing and off-target effects [111], considerable efforts were undertaken to validate candidates of important host factors, by gene silencing and mutant analysis in adult flies and confirmation of results in mosquito cells [110,113,117,118,119]. Furthermore, it was regularly observed that host factors in *Drosophila* cells were conserved in mammalian (human) cells [114,115,116,119,120,121,122]. The results of the screens with respect to the (validated) identification of both new resistance mechanisms (VRFs, “antiviral genes”) as well as (often virus-specific) cellular processes that are limiting to viral infection (VSFs, “proviral genes”) are summarized in Table 1. Below follows a more detailed discussion of the major themes that have emerged from the large-scale RNAi screens for VSFs and VRFs.

#### 3.2.1. No Identification of RNAi Machinery Components

Although considered a major antiviral pathway, it is striking that no core RNAi factors (Dcr-2, Ago-2) were discovered in the genome-wide RNAi screens. This could partially be explained by the set-up of the assay that prevents the complete knockdown of genes that are necessary for the silencing process. Nevertheless, targeted “RNAi-of-the-RNAi” screens have been performed successfully to identify core and associated RNAi factors in cultured cell lines [72,73,123].

While some antiviral factors were found to be associated with the RNAi machinery, no clear evidence was found that their antiviral action was (mainly) mediated through the RNAi process. While loss of Ars2 leads both to a decrease in siRNA-mediated silencing and an increase in viral infection, functions of Ars2 were also uncovered in miRNA-mediated silencing (which is not considered a canonical antiviral pathway in *Drosophila*) [124]. Ars2 also interacts with components of the nuclear cap-binding complex in addition to Pasha (co-factor of Drosha, miRNA pathway) and Dcr-2. The role of Ars2 in antiviral defense therefore could be much more complex than just by acting as a cofactor in Dcr-2 processing.

The DEAD box RNA helicase Rm62 was identified as an antiviral factor of RVFV infection in the RNAi screens [114]. In another study, Rm62 was also shown to bind Ago-2 and control siRNA silencing [61]. However, studies in mammalian cells indicate that DDX17, the homolog of Rm62, can interact with the intergenic region of RVFV that resembles a miRNA hairpin [113]. Since DDX17 also interacts with enzymes in the canonical mRNA degradation machinery, it was proposed that Rm62/DDX17 may function as an RNA sensor of RVFV infection to facilitate (RNAi-independent) degradation [114].

Finally, an ancient antiviral role was demonstrated for Drosha, the RNase III component of the microprocessor complex that processes primary miRNA transcripts in the nucleus [122]. However, Drosha is proposed to act as an antiviral factor through direct recognition/processing of RNA stem loops, conform to its ancestral function, independently of the antiviral RNAi machinery (Dcr-2, Ago-2) or its role in primary miRNA processing, that both later evolved.

#### 3.2.2. Virus-Specific VRFs (Antiviral Genes) and VSFs (Proviral Genes)

Factors that specifically restrict bunyavirus (RVFV, LACV) infections include three genes (*Dcp2*, *LSM7*, *Me31B*; Table 1) that are involved in the process of de-capping of host mRNAs [125]. The de-capping process plays an important role in the bunyaviral infection cycle since 5′-caps for bunyaviral mRNAs are acquired by “cap-snatching” the 5′-ends of cellular mRNAs. Viruses that do not “cap-snatch” the 5′-end of host mRNAs, such as VSV, DCV and SINV, were not affected by the knockdown of the de-capping factors. Interestingly, sensitivity to knockdown of cell cycle genes (Table 1) was explained by the preferential cap-snatching from cell cycle mRNAs [125].

Genome-wide RNAi screens resulted in the identification of the cellular receptor required for SINV infection, i.e., Malvolio (Mvl) or divalent metal ion transporter natural resistance-associated macrophage protein (dNRAMP) (Table 1; [117]). Further studies demonstrated that expression of dNRAMP is regulated by genes in the ER-associated protein degradation (ERAD) pathway (dSEC61A, dVCP) and the proteasome (dPSMD11) (Table 1; [119]). On the other hand, dNRAMP was completely dispensable for WNV and VSV infections.

Translation of DCV dicistroviral proteins is initiated by internal ribosomal entry sites (IRES) in the viral mRNAs. RNAi screens established that DCV replication is specifically inhibited following knockdown of ribosomal proteins (RpS6, RpL19; Table 1). The requirement for expression of high levels of the translation machinery was not observed for VSV which uses a 5′-cap initiated translation mechanism [126]. Interestingly, in yeast, ribosomal protein RpS25, which is not an essential protein, is required for interaction of the (intergenic region (IGR)) IRES from CrPV with the 40S ribosomal subunit and subsequent translation [127].

When an RNAi screen was performed targeting 16 ribosomal proteins, previously identified to interact with the core RNAi machinery (Dcr-2, Ago-2, R2D2), depletion of RACK1 was found to decrease significantly the viral titers of the dicistroviruses CrPV and DCV, but not of FHV (*Nodaviridae*) and VSV (*Rhabdoviridae*) [128]. While initially identified as an adaptor protein interacting with a variety of signaling molecules (e.g., protein kinase C), RACK1 was later identified as a component of the 40S ribosome subunit. Also silencing of the eIF3j subunit of the translation initiation factor eIF3 interfered with dicistrovirus infection. Although RACK1 is also involved in miRNA function, its major inhibitory action on dicistrovirus replication seems to be by acting as a scaffold protein to recruit signaling pathway components to regulate translation at the (5′- but not the IGR) IRES, for instance through modification of eIF3j [128].

Interaction with Toll-7 and its subsequent activation of autophagy was identified as an antiviral pathway protecting against viruses with a negative strand RNA genome such as VSV and RVFV [26,101,129]. Antiviral Toll-7 signaling was independent of the Toll signaling components MyD88 and the NF-κB transcription factor Dif [26]. Depletion of the insulin signaling component Akt, which increases autophagy, inhibited VSV replication [101]. The Toll-7/autophagy pathway was not involved in protection against viruses with a positive RNA genome such as DCV, FHV and SINV [101,129].

The screening of a library of biologically active molecules identified several drugs that restrict RVFV infection [121]. Drugs inhibiting ion pumps, the cytoskeleton and protein kinase C (PKC) as well as apoptosis inducers were significantly overrepresented among the compounds that attenuated viral infection. Targeted screens identified the specific requirement of the PKC epsilon isozyme (PKCε) at an early step in the infection cycle. Silencing of PKC98e (PKCε homolog) in adult flies resulted in increased sensitivity to RVFV infection, while no effects were observed on DCV infection [121].

When a targeted RNAi screen for silencing of genes involved in glycerophospholipid metabolism was performed, five genes were identified that are involved in FHV RNA replication [130]. The specific requirement for genes involved in phosphatidylcholine synthesis can be explained by the association of the FHV replication process to the outer mitochondrial membranes and the identification of the viral RNA-dependent RNA polymerase as a lipid-interacting protein.

Following a CRISPR screen in human cells, subsequent validation experiments by RNAi in *Drosophila* cells confirmed the requirement for the signal peptidase complex for proper cleavage of WNV and DENV structural proteins and secretion of viral particles [116]. At least in human cells, silencing of signal peptidase complex subunits has no effect on alphavirus, bunyavirus or rhabdobvirus infections, indicating a specific requirement for infections by flaviviruses.

Although not identified in RNAi-based screens, another antiviral mechanism can be mentioned that is specific for FHV. FHV was demonstrated to be a cardiotropic virus in *Drosophila* that is controlled by a mechanism that involves ATP-sensitive potassium (K_ATP_) channels [131,132]. Genetic interactions occur between potassium channel and *ago-2* mutations leading to the proposition that RNAi can be regulated by potassium ions, as is observed for other immune response mechanisms in mammals. By contrast, modulation of activity of K_ATP_ channels did not affect infections by DCV.

DsRNA-binding proteins are another example of factors that can contribute to antiviral immunity. Disconnected Interacting Protein 1 (DIP1) has a role in tRNA processing and maturation but has also antiviral activity against DCV infections, in contrast to DXV infections [133].

#### 3.2.3. Other Host Factors Required for Viral Infection (VSFs)

A common pathway for viruses to enter cells is endocytosis which is reflected by the sensitivity of DCV and VSV to the knockdown of the small GTPase Rab5, a regulator of endosomal trafficking (used as positive control in RNAi screens [126,134]).

A genome-wide RNAi screen using *Drosophila* cells also identified several classes of host factors that are required for DENV infection (proviral genes), involved in processes such as endocytosis, vesicular transport, vacuolar identification, ER function, unfolded protein response and RNA metabolism (Table 1; [120]). It is not clear whether the identified VSFs are specifically required for DENV (flavivirus) infection since no comparisons were carried out with other virus infections.

All viruses with a positive strand RNA genome are known to undergo replication in association with membranes of the infected cells. Such targeted localization is thought to provide advantages such as efficient separation of different viral functions (replication, transcription, translation) and protection from immune recognition. The requirement to associate with cellular membranes was revealed during a whole genome RNAi screen for identification of proviral genes during DCV infection [134]. More specifically, the COPI coatamer complex, responsible for retrograde transport of recycled proteins from Golgi to ER, and fatty acid metabolism (the enzyme fatty acid synthase and the master transcriptional regulator of lipid homeostasis SREBP) were identified as limiting factors/processes during DCV infection. However, whether infections of other insect RNA viruses and arboviruses were equally sensitive as DCV to the identified genes/processes was not directly investigated.

At later stages of viral infection high amounts of structural (capsid) proteins are required indicating the need for an efficient translation process. A limiting factor in this process could be the occurrence of stalled ribosomes as a consequence of translation errors. The pelo/Hbs1 complex, which recognizes and resolves stalled ribosomes as part of a quality control process of protein translation, was identified as required for efficient replication of a diversity of viruses such as CrPV and DCV, the birnavirus DXV and the DNA virus IIV-6 [135]. Initially identified as a VSF in a screen of a collection of mutant *Drosophila* flies, the requirement was subsequently shown by RNAi knockdown in S2 cells. The interference of *pelo* with viral replication did not occur through inhibition of RNAi or the induction of the JAK/STAT pathway and Vago ([135]; see also below in Section 3.4).

#### 3.2.4. Broad Range Antiviral Defense Programs

Arbovirus infection of *Drosophila* cells results in the induction of an antiviral program that is transcriptionally complex and includes components of the immune pathways JAK/STAT, Imd, Toll, RNAi and autophagy [118]. Part of the antiviral transcriptional program is affected by the transcriptional pausing machinery (negative elongation factor NELF and positive elongation factor P-TEFb; Table 1) which is responsible for a rapid response to virus infection. Increases in infectivity following suppression of transcriptional pausing are observed for different arboviruses such as KUN, RVFV, VSV and SINV, but also the *Drosophila*-specific virus DCV [118]. Further studies demonstrated the involvement of the nucleoporin Nup98, that also can function in transcription, and the transcription factor FoxK in the induction of the antiviral program [136,137], while also an antiviral function could be demonstrated for some of the induced genes such as the splicing factor B52 which was analyzed in more detail [137].

Targeted RNAi screens also identified the receptor tyrosine kinase PVR (*Drosophila* PDGF/VEGF receptor) and the ERK pathway as a broad-acting antiviral defense mechanism (restricting DCV, VSV, SINV and DENV; Table 1) [112,113]. Interestingly, in the intestinal epithelium this pathway is dependent on the gut microbiota that act by priming the gene encoding Pvf2, a ligand for the PVR receptor kinase, to be able to respond rapidly following viral infection through a mechanism that also involves transcriptional pausing [112]. The antiviral immune mechanism in the gut epithelium is also dependent on the Imd pathway and will be discussed in greater detail below.

Other broadly acting host factors that restrict arbovirus (two strains of WNV, DENV, SINV, RVFV and VSV) infection in S2 cells and were subsequently validated in adult flies and mosquito cells, include the Tip60 histone acetyltransferase complex, involved in chromatin modeling, and dXPO1, a karyopherin protein that exports proteins and RNAs from the nucleus to the cytoplasm (Table 1; [110]). An antiviral role was also demonstrated for the enzyme aldolase, for which the nuclear transport of its mRNA was shown to be dependent on dXPO1.

It is not surprising that also the (non-RNAi) RNA degradation machinery was identified as an antiviral defense mechanism acting against arboviruses with both positive (SINV) and negative strand (VSV, RVFV) ssRNA genomes [115]. Viral RNAs often have distinctive features such as dsRNA structures, 5′-triphosphates and short or absent poly(A) tails and such “aberrant” RNAs can be bound by RNA-binding cofactor complexes for subsequent targeting to the RNA degradation machinery. In targeted RNAi screens both the 3′-to-5′ RNA exosome and components of the exosome cofactor complex TRAMP (Trf4/5–Air1/2–Mtr4 polyadenylation) were implicated in antiviral defense (Table 1; [115]). On the other hand, no factors of the other exosome cofactor complexes (Ski, NEXT) were found to be involved, revealing some specificity in the viral RNA recognition process. In contrast, as noted earlier, a 3′–5′ exonuclease and RNA-binding proteins were also identified as proviral during RNAi screens of DENV flavivirus infections (Table 1; [120]). This difference may be caused by the experimental set-up of the RNAi screens which can be biased to the identification of proviral genes (sensitized at high infection rate) versus antiviral genes (sensitized at low infection rate) [110].

### 3.3. Involvement of Innate Antimicrobial Immune Pathways (Toll and Imd)

Innate immunity against bacterial and fungal infection classically is divided between humoral and cellular immunity. Most cellular immune responses, such as phagocytosis, melanization and encapsulation, are mediated by the hemocytes while secretion of AMPs following pathogen infection is carried out by fat body tissue [138].

The Toll and Imd pathways are both NF-κB-related pathways that are activated by Gram-positive bacteria/fungi or Gram-negative bacteria, respectively. In the case of the activation of the Toll pathway, interaction of pathogen-associated molecular patterns (PAMPs) with pathogen recognition receptors (PRRs) results in a proteolytic cascade leading to the processing of Spätzle. The Toll signaling pathway is activated following the binding of Toll by Spätzle and culminates in the induction of AMP gene expression by the NF-κB transcription factor Dif [27]. It is noted that a highly similar Toll pathway, but with a different NF-κB transcription factor, Dorsal, acts to mediate dorsal-ventral patterning of the early embryo [139]. Activation of the Imd pathway is also achieved after sensing PAMPs derived from Gram-negative bacteria and results in the activation of the NF-κB transcription factor Relish by a double-branched pathway, i.e., phosphorylation of Relish by the IκB kinase (IKK) complex and cleavage of phosphorylated Relish by Dredd caspase [27].

Both Toll and Imd pathways have also been shown to be involved in antiviral innate immunity in *Drosophila*. However, not all genes associated with bacterial or fungal infection seem to be functional, indicating the existence of “non-canonical” Toll- and Imd-related pathways during antiviral defense.

Injection of DXV (*Birnaviridae*) in the hemocoel of adult flies results in the induction of AMP genes that are associated with both Toll and Imd activation [140]. On the other hand, only particular mutations in the Toll signaling pathway were shown to affect viral titers and virus-induced mortality. Activation of the antimicrobial pathways may not be through direct recognition of viral PAMPs but is suggested to occur indirectly through damage of infected tissues. Cellular debris released during cell rupture could act as “damage-associated molecular patterns” (DAMPs) to activate the antimicrobial signaling pathways [82,140,141,142]. AMPs do not protect directly against virus infection since over-expression in transgenic flies does not confer protection against DXV [140]. Significant induction of some AMP genes was also observed after Sigma virus (MelSV) infection [143].

In contrast to DXV and Sigma virus, injection of the dicistrovirus CrPV in *Drosophila* adult flies did not result in an increase in AMP gene expression [144]. Weak or no AMP induction was also observed during infection with DCV (*Dicistroviridae*; related to CrPV) [145,146]. On the other hand, mutations in one of the branches of the Imd signaling pathway (*dTAK1-kenny-Ird5*; leading to phosphorylation of Relish), *relish* (*rel*) and the gene encoding peptidoglycan recognition protein-LC (PGRP-LC; a PRR in the Imd pathway) resulted in increased sensitivity to CrPV infection. However, *imd* itself was dispensable for the response against CrPV infection, as well as *dFadd*, acting in the second branch of the canonical Imd pathway that leads to proteolytic cleavage of Relish. Thus, the different branches of the Imd pathway may contribute differently to the antiviral response [144]. Virus-specific effects are also apparent since *relish* mutations do not show a phenotype during DCV or DXV infections [140,147]. Furthermore, *Dif* (Toll pathway) and *key* (Imd pathway) did not have an impact on infectivity of DCV after injection [98].

In flies that are transgenic for an inducible SINV replicon (inducible viral replication in the absence of production of virions), both the JAK/STAT (see Section 3.4) and the Imd pathway (but not the Toll pathway) were found to be implicated in antiviral resistance [148,149]. In this case, both branches of the Imd pathway (Relish phosphorylation and cleavage) were shown to be involved but not the (bacteria-specific) PRRs PGRP-LE and -LC. The induction of AMPs was also observed and partly interpreted as a prophylactic immune response, aimed at the prevention of secondary bacterial infections [148]. Interestingly, the direct involvement of the AMPs Attacin C and Diptericin B (encoded by *attC* and *dptB*, respectively) in the control of SINV replicons and SINV viral titers was also demonstrated [149]. Knockdown of *dptB* in SINV replicon flies induced mortality at the early pupation stage [149]. While a role for Dicer-2 was identified to restrict SINV RNA replication by the RNAi mechanism, the induction of Relish-mediated transcription was found to be independent of Dicer-2 [148]. Thus, Dicer-2 does not function as a viral sensor in the activation of the Imd pathway, in contrast to the JAK/STAT pathway ([147,150]; see Section 3.4). The antiviral action of the Imd pathway (but not the Toll pathway) was also confirmed after injection of SINV virus in the hemocoel and tissue-specific knockdown of Relish showed a major requirement for this NF-κB transcription factor specifically in the hemocytes [148]. Induction of *relish* and the immune gene *Thiol-ester containing Protein II* (*TEPII*) was also observed after SINV infection of S2 cells [118,151].

Previous studies mentioned in this section were carried out after the administration of the virus to adult flies by injection, which is not a natural route of infection. Natural virus infections in *Drosophila* occur by vertical transmission (Sigma virus or DMelSV) or by feeding (DCV, Nora virus). In the case of DCV, however, differences in tissue tropism between the two methods of virus administration (injection versus feeding) were only observed during the early stages when infection is more widespread after injection than after feeding [96]. Nevertheless, the gut epithelial barrier must be considered as a formidable obstacle against pathogen infection and it is expected that specific defense mechanisms are associated with the gut epithelium. That route of infection can have a large impact on innate immunity was demonstrated by the observation that mutations in the Toll pathway (*Toll* (*Tl*), *spätzle* (*spz*), *tube* (*tub*) and *pelle* (*pll*)) result in increased susceptibility to oral but not systemic infection by DCV, CrPV, Nora virus and FHV [96]. Interestingly, resistance to oral viral infection requires Dorsal as NF-κB transcription factor and not Dif which is required to resist bacteria and fungi. The difference in viral titers between wild-type flies and *pll* mutants is comparable with the difference observed for RNAi mutants, underscoring the importance of the Toll pathway. However, also here the Toll pathway may not be activated directly by the virus but secondarily through a secreted factor since activation of the pathway (AMP reporter gene expression) can occur in cells not infected by the virus [96]. As already suggested, tissue damage by viruses can also result in activation of the Toll pathway [142]. In such case, activated Spätzle may act as a protective secreted factor (“cytokine”) to guard against the spread of viral infections.

Interestingly, excessive activation of the Imd immune pathway can result in increased mortality caused by viral infections. Such reduced viability was observed in *diedel* (*die*) mutants of *Drosophila* after infection with SINV virus (but not with other RNA viruses such as VSV, DCV, CrPV and FHV) in the absence of increases in viral titers [152]. Diedel is a small (12 kDa) secreted factor produced in the fat body that is highly induced following SINV and VSV infection (while only SINV-induced mortality is sensitive to *die* mutations). Induction involves a non-canonical Toll pathway that is dependent on the NF-κB factor Dif but not on the adaptor protein MyD88. Transcriptome analysis reveals increased expression of immune-related genes in *die* mutants in the absence of infection that can be correlated with reduced viability. During SINV infection, genes in the Imd pathway are much more strongly induced in *die* mutants which was implicated in the pathological effects since the pathology phenotype was rescued in double mutants of *die* and genes in the Imd pathway (*key*, *imd*) [152]. Diedel is therefore considered an important regulatory point to “dampen” the immune response and to protect against tissue damage. The necessity for tight control of immune pathways to prevent pathological damage from excessive activation has also been reported for the JAK/STAT pathway and is related to the concept of virus “tolerance” rather than “resistance” ([82,153,154]; see also Section 3.4).

As already mentioned in Section 3.2.4, RNAi screens identified a signaling circuit involving PVR, its ligand Pvf2 and ERK signaling, that controls DCV and arbovirus (including SINV) infections [112]. When these studies were extended to the gut epithelium, it was observed that priming by the microbiota was necessary for robust induction of Pvf2 in the intestinal epithelium following viral infection. The mechanism of priming of the microbiota however requires components of the Imd pathway (Imd, Tak1) and the NF-κB factor Relish, but not the Toll pathway [112], which seems in contrast to the previously mentioned study [96]. Both studies also found different effects of antibiotics on the antiviral defense mechanism (no effect: [96]; proviral effect: [112]). Further studies are necessary to explain the different experimental outcomes and to clarify the roles of both Imd and Toll pathways in antiviral defense.

Polydnaviruses (*Polydnaviridae*; [155]) constitute non-replicative viral particles that are produced in the female reproductive organs of parasitoid hymenopteran wasps and co-injected with the eggs in (mainly lepidopteran) insect hosts [156]. Also, *Drosophila* has functioned as a model to study parasitoid wasp infection [157], and to clarify the role of polydnaviruses in the suppression of the immune response, particularly with respect to the inhibition of NF-κB signaling by polydnaviral Ankyrin (Vankyrin) proteins [158,159,160]. However, in this case, the major purpose for suppression of the immune response is the survival of the parasitoid eggs and larvae and not the facilitation of viral replication. However, replication-defective polydnaviruses have evolved strategies for preferential translation of viral mRNAs in host cells [161]. Whether polydnaviruses encode genes that suppress RNAi has not been reported.

### 3.4. JAK/STAT Pathway

The major components of the JAK/STAT pathway comprise three Unpaired (Upd1, Upd2, Upd3) ligands, the cytokine receptor Domeless (Dome), the Janus Kinase Hopscotch (Hop) and the signal transducer and activator of transcription Stat92E [27]. The pathway is typically activated in a paracrine fashion by binding of the ligands to the Dome receptor. Production of the ligands occurs after recognition of infection through PRRs and pathways that are largely unknown.

Injection of the *Drosophila*-specific virus DCV in adult flies resulted in transcriptional induction of approximately 150 genes that have a distinct profile compared to bacterial or fungal infections [146]. One of the genes with a unique virus-specific induction profile, *virus-induced RNA 1* (*vir-1*), that encodes a small protein without clear structural motifs, was studied in more detail. Genetic analysis shows that *vir-1* induction by DCV is regulated by the JAK/STAT pathway and binding sites for the STAT transcription factor were identified in the promoter of *vir-1*. Interestingly, *vir-1* expression occurs in tissues that are different from the major sites of DCV infection, indicating a secondary response possibly initiated by a secreted factor (“cytokine”) produced by virus-infected tissues [146]. The function of *vir-1* in the antiviral response remains unidentified since over-expression of *vir-1* in transgenic flies does not protect against viral infection. It is also noted that none of the genes of the RNAi machinery were induced following DCV infection [146].

The role of the JAK/STAT pathway was confirmed in flies mutant for the JAK kinase Hopscotch, that showed higher mortality and increased viral titers after DCV and CrPV dicistrovirus infection, but not after infection with other RNA viruses (VSV, FHV, DXV, SINV) and the DNA virus IIV-6 [34]. Thus, while the RNAi pathway is broadly effective against many virus infections, the JAK/STAT pathway seems to be more restricted to dicistrovirus infections. Specific responses against viral infections are also apparent in genome-wide microarray studies where different sets of genes are induced by DCV, SINV and FHV infections [34]. However, as already mentioned in the previous Section 3.3, using another approach, the Stat92E transcription factor was shown to restrict the SINV replicon in transgenic flies [148]. A later study confirmed that a majority of genes that are upregulated in flies with a SINV replicon, had STAT Relish-binding sites in their promoters and are regulated by STAT and Relish [149].

In addition, Vago, a secreted protein of 160 amino-acids with a conserved single “von Willebrand factor type C” (VWC) domain [150], was also induced by DCV and SINV (but not FHV) infection [147]. The antiviral effect of Vago is required in the fat body and evidence indicates that Vago is directly induced by virus infection in that tissue (in contrast to *vir-1*). Induction of Vago expression was independent of the three main immune signaling pathways (Toll, Imd, JAK-STAT) but required an intact DExD/H-box helicase domain of Dicer-2 [147]. Thus, Dicer-2 may function as a viral sensor to induce innate antiviral immune defense pathways, similar to the RIG-I-like receptor (RLR) helicases in mammals, to which Dicer-2 is evolutionary related with respect to its DExD/H-box helicase domain. The relationship between *vir-1* and *Vago* remains unclear since *vir-1* remains fully inducible by virus infection in *vago* (as well as *dcr-2*) mutants. In mosquito cells, on the other hand, it was demonstrated that mosquito Vago could activate the JAK/STAT pathway to induce *vir-1* expression [150]. Up to 13 factors with a single VWC domain can be identified in the *Drosophila* genome and their role may not be limited to antiviral defense but likely extends to nutritional control and environmental stress [162]. Whether the particular Vago protein that is induced by DCV and SINV infection in *Drosophila* has a specific function in antiviral defense or may also be involved in the regulation of other stress-related processes needs further experimental verification.

The genes that are induced by dicistrovirus infection in *Drosophila* also include the ligands for the Dome receptor, i.e., Unpaired 2 and 3 (Upd2 and Upd3) [34]. The induction of Upd2 and Upd3 was suggested to be an indirect response [35] since it is known to occur after tissue damage and released cell debris, for instance after septic injury [163] but possibly also after viral infection. The observation that *vir-1* is not induced by inactivated virus or dsRNA but requires viral replication [164] is also consistent with an indirect response to “danger signals” rather than direct activation of a pathway through specific interaction between viral PAMP and host PRR [165].

A recent study indicated that over-expression of the JAK/STAT pathway can lead to increased pathology and mortality during infection by RNA viruses (DCV, CrPV, DXV and FHV) but not by a DNA virus (IIV-6) [153]. The epigenetic regulator and H3K9 methyltransferase G9a was found to cause the “dampening” of the JAK/STAT-mediated antiviral response and therefore involved in the mechanism of “tolerance” during RNA virus infection. In contrast to resistance, involved in the control of viral titers, tolerance is associated with the limitation of damage that can occur during viral infection [28,82,154]. Tight control of JAK/STAT signaling is necessary to achieve an efficient antiviral immune response since both repression and over-stimulation can result in increased mortality [34,153]. As already discussed above (Section 3.3), negative control mechanisms were also reported to prevent excessive activation of the Imd pathway and its possible associated immunopathology [152,166].

### 3.5. c-Jun N-terminal Kinase (JNK) Pathway

A strong transcriptional activation of the JNK pathway was also reported after intrathoracic injection of DCV, which included pathway components (*Hemipterous*, *Gadd45*, *Jra*, *Kay*) as well as downstream targets (*Puckered* and *Rab-30*) [153]. In addition, promoter regions of differentially expressed genes were enriched for binding sites of the JNK signaling cascade transcription factor AP-1. During RNAi screens in S2 cells, however, no association with the JNK pathway was found [113]. Inhibition of JNK signaling also did not impact viral infection in the intestine after feeding [112].

### 3.6. Transcriptional Programs Induced by Viral Infection

Analysis of microarray hybridizations and next-generation transcriptome sequencing has revealed the complexity of the transcriptional programs of virus infections in cell lines or adult flies. Transcriptional responses are considered to be virus-specific although this is obscured by the poor reproducibility of transcriptome data [35]. Virus specificity of transcriptional responses is probably also strongly affected by their different tissue tropism [28], in addition to type of genome or replication strategy.

An overview of published transcriptome studies is presented in Table 2. Changes in gene expression however do not reveal function and require additional experimentation to establish the (negative or positive) role in the infection process. This can be achieved by RNAi-mediated silencing or mutant analysis as discussed in many different examples in previous sections. This has resulted in the identification of host factors necessary for viral infection (proviral genes) as well as the involvement of the immune system such as the JAK/STAT pathway and RNAi (antiviral genes). From the large number of genes that are altered upon viral infection (typically from about 100 to several hundred in each transcriptome study) only for a low proportion their role in the infection process could be confirmed, mainly because of the disproportionate effort to carry out the validation process.

Differential expression of genes is not necessarily a direct consequence of virus detection. Very few viral sensors (PRRs) have been identified such as the helicase domain of Dcr-2 [147] and Toll-7 [26]. Virus infection often results in the generation of secondary transcriptional responses in other tissues that are mediated by systemic signals [147]. Activation of the JAK/STAT pathway (*vir-1*, Turandot proteins) likely is a secondary response caused by tissue damage and stress signals associated with viral replication [35,153,167]. Unique responses can also be generated by pathological effects by the virus, for instance during intestinal obstruction caused by DCV infection [168].

RNAi-related genes are usually not identified among the virus-induced genes [146]. This could be related to the relative late time points of data collection post infection in most studies (Table 2) since Dcr-2 was identified among the early genes (4 h post-infection (hpi)) induced after VSV infection in S2 cells [118].

As already indicated above, comparison of transcriptome data has also revealed their poor reproducibility, even if infections were carried out with the same virus. As discussed by Marques and Imler [31], this could be caused by (among others) differences in the experimental system (cell lines versus adult flies) and infection routes (systemic versus oral), shortcomings of experimental procedures (for instance, off-target effects in RNAi), heterogeneity in genetic background of *Drosophila* strains, polymorphisms in host restriction factors and the unknown occurrence of persistent infections (other viruses, *Wolbachia*). Infection by natural (*Drosophila*-specific viruses) and non-natural pathogens (arboviruses) are expected to give different responses because of (absence of) co-evolution of the pathogen with the host. Infections should be investigated in the right context because of the occurrence of virus-specific and tissue-specific mechanisms.

### 3.7. Secreted Antiviral Factors and the Systemic Response

In mammals, the interferon system is a powerful system that is capable to restrict most viral infections even in the absence of adaptive immunity [169,170]. Interferons are secreted factors that are produced after detection of viral PAMPs (e.g., dsRNA) by PRRs (e.g., Toll-like receptor 3) [171]. Interferons subsequently stimulate the expression of antiviral interferon-stimulated genes through activation of the JAK/STAT pathway [172]. Secreted factors and signals that induce a systemic response are also produced during viral infections in *Drosophila* but how much such an “interferon-like” system exists in invertebrates remains under debate [173]. Several secreted factors that play a role in the regulation of the antiviral response in *Drosophila* were already discussed and include Vago (Section 3.4), Pvf2 (Section 3.2.4 and Section 3.3), Diedel (Section 3.3) and AMPs (Section 3.3). It is noted that the Unpaired ligands of the JAK/STAT pathway are induced during dicistrovirus infections, possible through stress signals and tissue damage [35], which can also be interpreted as the activation of a systemic antiviral response. Similarly, protease activity associated with necrosis or abnormal apoptosis of virus-infected cells could activate the Spätzle ligand and induce the Toll pathway [96,142].

MALDI-TOF mass spectrometry was also used to analyze secreted factors in the hemolymph following DCV infection which resulted in the identification of Pherokine-2 (Phk-2; [145]). Phk-2 is related to a previously characterized odorant/pheromone-binding protein expressed in the antennae and its developmental profile also suggests a role in tissue remodeling during metamorphosis. Over-expression of Phk-2 in transgenic flies does not result in increased protection against DCV infection [145].

### 3.8. Cellular Responses against Viral Infections: Phagocytosis, Apoptosis and Autophagy

Cell-mediated immunity in insects includes phagocytosis, nodulation, encapsulation and melanization and is primarily mediated by the hemocytes or blood cells [174]. The majority of blood cells constitute the macrophage-like plasmatocytes (90–95% of hemocytes in *Drosophila*) that are specialized in the engulfment and degradation of cellular debris, debris and invading pathogens [175].

The involvement of cellular immunity in antiviral defense was dramatically demonstrated in experiments of genetic ablation of hemocytes or inhibition of their phagocytotic capacity by injection of polystyrene or latex beads [144,176]. Virus-specific effects were observed since cellular immunity was required for resistance against CrPV, VSV and FHV but not against SINV or IIV-6. In the case of DCV, phagocytosis was required to control infection at high doses [30] but was dispensable at low doses [176]. During CrPV infection, hemocytes become depleted which is associated with the viral dose and progression of infection [144].

The requirement for phagocytosis seems to be correlated with the induction of apoptosis during viral infection. CrPV, DCV and FHV trigger apoptosis in the S2 cell line and apoptotic bodies can be subsequently phagocytosed by plasmatocytes from adult flies [176] or l(2)mbn cells, a *Drosophila* larval hemocyte-derived cell line [30]. In the latter study of DCV infection, it was shown that phagocytosis preferentially targets apoptotic cells and that it involved the recognition of specific features of apoptotic cells (e.g., exposed phosphatidylserine glycerophospholipid on the cell surface) by the engulfment receptors Draper and integrin βν of the hemocytes [30]. Related to the importance of apoptosis/phagocytosis to control DCV infections only at high doses, it was argued that other defense mechanisms (e.g., RNAi) can reduce the levels of viral replication and damage to the point where they are not pro-apoptotic (as seems to be the case for SINV). Nevertheless, the importance of apoptosis to control viral infections was demonstrated by recombinant SINV viruses that express the pro-apoptotic gene *reaper* [177]. Viruses expressing a pro-apoptotic gene were selected against to establish persistent infections in mosquitoes, presumably because of the strong antiviral effect of the pro-apoptotic gene [178].

In another study, injections of the RNA virus FHV or the DNA virus *Autographa californica* multiple nucleopolyhedrosis virus (AcMNPV, *Baculoviridae*) in larval or adult *Drosophila* resulted in the rapid (1–2 hpi) induction of pro-apoptotic “RHG” (*reaper*, *hid*, *grim*) genes, mainly in fat body tissue [179]. The early response in animals is in contrast to the late response in *Drosophila*-derived DL-1 cells in vitro (24–36 hpi), where it is not associated with major effects on viral proliferation (see further below). The rapid induction of RHG genes in fat body was followed by apoptosis at 2.5 hpi and resulted in blockage of viral gene expression and proliferation. Genetic analysis indicated that induction was dependent on the irradiation responsive enhancer region of the RHG gene cluster and the transcription factor P53, as well as caspase (Dronc) activation [179]. It is noted that the induction of genes related to apoptosis (e.g., caspase genes) was also observed in other transcriptome studies, e.g., [146].

The role of phagocytosis in antiviral defense against DCV infection was also investigated in *Drosophila* cell lines S2 and DL-1. Knockdown of the GTPases Rab5 (early phagosome) and Ran (mostly reported to be an essential player in nuclear transport but with additional role in phagocytosis) resulted in lower levels of phagocytosis of DCV and was associated with higher viral titer [180]. Studies of infection of S2 cells with white spot syndrome virus (WSSV, *Nimaviridae*, a shrimp DNA virus that does not replicate in *Drosophila*) showed engulfment but no degradation of WSSV indicating mechanisms to avoid phagosome maturation and lysosome-mediated degradation [181]. Activation of the Toll or Imd pathway by lipopolysaccharide or peptidoglycan was sufficient to target WSSV to the lysosomes. Gene expression profiling followed by functional studies indicated a role for *dally* (*division abnormally delayed*, encoding a cell surface receptor) and the associated Wnt signaling pathway in phagocytosis of WSSV virus [181].

The mechanism of induction of apoptosis by viral infection was analyzed in greater detail in DL-1 cells with respect to the RNA virus FHV and the DNA virus AcMNPV (to be discussed in the paragraphs below). As mentioned earlier, virus-induced apoptosis in the cell line is a late event and is not associated with protection against viral multiplication [182], in contrast to infections in animals. It is speculated that the early mechanism of stress-induced cell death is lost during the selection process for establishment of permanent cell lines which points to a limitation in the use of cell lines to study mechanisms of antiviral defense [179].

In DL-1 cells, for both FHV and AcMNPV infections, apoptosis is induced following a depletion of the cellular anti-apoptotic factor DIAP1 (Drosophila inhibitor-of-apoptosis 1) [182,183]. The reduction of DIAP1 results in the activation of (initiator Dronc/Dark and effector Drice) caspase activity followed by cytolysis and membrane blebbing, being hallmarks of apoptosis. General shutdown of cellular protein synthesis is considered to contribute to DIAP1 depletion and the induction of apoptosis during FHV infection [182].

While the baculovirus AcMNPV is mainly known to infect lepidopteran insects and cells, it can also support DNA replication and induce apoptosis in DL-1 cells [184]. The use of DL-1 cells and the extensive knowledge of apoptosis pathways in *Drosophila* have contributed significantly to the understanding of the mechanism by which baculoviruses induce and simultaneously prevent (by production of apoptosis inhibitors) the process of apoptosis. These pathways likely also apply to infections of other DNA viruses that are specific to *Drosophila* but have not been studied yet. In summary, the pathway is initiated following viral DNA replication in the nucleus which triggers the DNA damage response and the activation of the phosphatidylinositol 3-kinase-like kinases ATM and ATR. Phosphorylation of the histone 2A variant H2AX is considered crucial for the amplification of the response and the recruitment of additional components in the pathway, including DNA repair factors [185]. Typical cellular responses are cell cycle arrest to allow DNA repair, and apoptosis to remove damaged cells. Upon activation of the DNA damage response, it can be speculated that the transcription factor P53, which is involved in the DNA damage response and also a regulator of the RHG gene cluster, can upregulate pro-apoptotic genes to destabilize DIAP1 which is central to the initiation of apoptosis as mentioned above.

While a role for phagocytosis/apoptosis in antiviral defense has become evident recently, less is clear about the involvement of another cellular process, autophagy. As already mentioned (Section 3.2.2), RNAi screens have identified a role for autophagy and *Toll-7* in the antiviral defense against viruses with a negative strand RNA genome such as VSV and RVFV [26,101,129]. Another study focused on the analysis of fly mutants of the autophagy gene *Atg7* and confirmed a role for autophagy in the control of VSV although the effects were considered mild [176]. By contrast, VSV infection levels were not affected in *Toll-7* mutants, in contrast to other studies [26,129]. No involvement of autophagy was found for the positive strand RNA viruses SINV, DCV, CrPV and the DNA virus IIV-6 while increased survival and decreased viral loads in *Atg7* mutants were observed for FHV (another positive strand RNA virus). FHV replication occurs at mitochondrial membranes and it is speculated that the removal of damaged mitochondria through autophagy (“mitophagy”) contributes to the success of FHV replication [176].

### 3.9. Heat-Shock Proteins and Stress

Transcriptome studies have revealed a role for the heat shock response in antiviral defense. Infection of S2 cells or adult flies with DCV results in the induction of 6 genes encoding various heat-shock proteins (Hsp70Ab, Hsp70Ba, Hsp22, Hsp23, Hsp26, Hsp27) [167]. Additionally, CrPV infections result in the induction of heat-shock protein genes but with delayed kinetics, while for a IIV-6 a clear heat-shock response is observed in S2 cells but not in adult flies. Furthermore, flies mutant for *Heat shock transcription factor* (*Hsf*) or transgenic flies with fatty body-specific knockdown of *Hsf* are more sensitive to viral infection [167]. The heat-shock response acts independently of RNAi or Jak-STAT pathway since no interference with RNAi-mediated silencing or antiviral gene induction is observed in *Hsf* mutants or knockdown animals. Finally, over-expression of Hsf or the heat-shock protein Hsp70 increases viral resistance. One possible mechanism for heat-shock proteins in antiviral defense could be their release from damaged cells and their subsequent action as “damage-activated molecular patterns” (DAMPs) to activate immune cells (hemocytes) as is observed in mammals [186].

While induction of heat-shock proteins is a late response (48 hpi) to CrPV infection in adult flies [167], studies in S2 cells have shown that CrPV inhibits the heat-shock response during early infection [24] which could be related to the rapid shutdown of host mRNA translation [187]. Although CrPV RNA and protein amounts are elevated at higher temperature, infectious virion production is nevertheless reduced by an unknown mechanism.

During infection of S2 cells with dicistroviruses (DCV and CrPV), extensive modulation occurs of stress granules and P-bodies, membrane-less organelles that contain RNA and protein complexes, while other types of poly(A)+ RNA granules are induced [188]. Inhibition of stress granule formation is considered important to keep viral RNA and proteins available for processing, translation and replication.

Since heat-shock proteins are molecular chaperones that mediate protein folding and re-folding, they are expected also to assist during specific molecular processes during viral infection. During FHV infection, the heat-shock protein Hsp90 is required for efficient translation of the RNA-dependent RNA polymerase (protein A) which becomes anchored in the external mitochondrial membrane during translation [189,190].

A more intimate relationship may exist between the heat-shock response and the RNAi machinery than expected. Besides their role in the cytoplasm during posttranscriptional gene silencing, the siRNA pathway factors Dcr-2 and Ago-2 also have a role in the nucleus to control the processivity of RNA polymerase II on euchromatic loci [191]. More specifically, knockdown of *ago-2* or *dcr-2* results in a significant increase in *Hsp70* transcripts under non-heat shock conditions. Chromatin immunoprecipitation and DNA fluorescence in situ hybridization experiments further demonstrate that both Ago-2 and Dcr-2 are integrated in the regulatory complex that causes RNA polymerase II pausing and play a role in the correct execution of the global transcriptional repression after heat-shock. Consistent with a role for the siRNA pathway in stress regulation, it was reported that *dcr-2* mutants were more sensitive to different types of stresses such as toxic chemicals, starvation and cold shock, and had a reduced lifespan [192]. Furthermore, abnormal lipid and carbohydrate metabolism was associated with loss of Dcr-2 and comparative proteomics revealed changes in expression of proteins associated with cellular metabolism, stress resistance, cell cycle [192].

During heat shock, Dcr-2 protein levels are reduced and dicing activity of long dsRNA substrates is diminished [193]. Furthermore, heat shock results in fragmentation of tRNAs which compete with canonical substrates of Dcr-2 such as long dsRNAs for processing. The RNA methyltransferase Dnmt2 was shown to be involved in the suppression of continuous tRNA fragmentation and consequently the recovery of the siRNA pathway after heat shock [193]. Interestingly, *Dnmt2* mutants show increased infection by the RNA viruses DCV and Nora virus [194]. Conversely, over-expression of Dnmt2 caused increased resistance to oral infection by DCV that was partly dependent on its methyltransferase activity. In *Dnmt2* mutants, the upregulation of immune response genes was muted. Binding of Dnmt2 to DCV RNA was also demonstrated and may contribute to virus control [194].

### 3.10. Wolbachia Infection

*Wolbachia* is a maternally inherited bacterial endosymbiont that resides within membrane-bound vacuoles of host cells and is widespread among arthropod species [138]. *Wolbachia*-infected flies are more resistant against infection by the RNA viruses DCV, Nora virus and FHV, while no effect is observed for infections with the DNA virus IIV-6 [195]. Protection by *Wolbachia* can occur very early in the infection process, at the level of the initial translation of incoming RNA and early replicative processes [52]. Furthermore, the antiviral resistance mechanism by *Wolbachia* occurs independent of the activation of the innate Toll and Imd pathways [96,176] as well as both miRNA- and siRNA-mediated RNAi [52]. Of interest is the observation that infections by *Wolbachia* can protect RNAi mutants (*dcr-2*, *ago-2*, *r2d2*) against infection by DCV and FHV [196]. Antiviral resistance is considered a cellular intrinsic mechanism that occurs in the absence of a transcriptional response in *Wolbachia* following viral infection, and may occur for instance through competition for intracellular resources and space or by remodeling the intracellular environment [52,197]. *Wolbachia* infections are also reported to stimulate the production of reactive oxygen species (ROS) which could further provide antiviral protection through stimulation of ERK signaling [198,199] (see also Section 3.2.4 and Section 3.3 for the role of ERK signaling). Protection by *Wolbachia* is variable, may depend on the *Wolbachia* strain and titer and can occur by both tolerance and resistance mechanisms [67,197].

## 4. Conclusions

Because of the wide range of genetic tools and online resources, the *Drosophila* model system has enabled dramatic advances in many areas of biological research [201], including the immune response against virus infections. Because research with *Drosophila* has acquired much more depth than with other insects, it can function as a benchmark to inspire similar research in other insects. In this review, information was gathered from the literature to evaluate the variety of defense mechanisms against virus infections in *Drosophila*. The basic purpose of this investigation was to provide a comprehensive overview of the multitude of antiviral defense strategies that include many non-RNAi pathways in addition to RNAi.

RNAi seems to be involved as an antiviral response to a certain degree against most, if not all virus infections. The importance of RNAi is most clearly illustrated by the specific generation of a siRNA pathway in somatic tissues of insects that is dedicated to defense against invading nucleic acids, and that is maintained separately from the miRNA pathway that regulates physiological and developmental processes [12,15]. The requirement for base-pairing prior to initiation of degradation provides great specificity and mechanisms for enhancement of efficiency have also evolved such as amplification via DNA forms and production of secondary siRNAs followed by systemic spread via exosomes [77,78]. On the other hand, efficiency of antiviral RNAi can be affected by physiological conditions such as nutritional status and stress [21,167]. Furthermore, during persistent viral infections, the RNAi pathway may be partially dismantled or may function in different ways that are not completely understood [87].

Complementary to RNAi, many other antiviral mechanisms exist that are often virus-specific. Viruses can trigger complex transcriptional responses during infection that overlap only in limited extent with each other (Table 2). Many genes identified in such transcriptional responses remain to be validated and for many factors it may also not be known whether they act provirally or antivirally. Similarly, genome-wide RNAi screens have revealed many resistance mechanisms that occur in the absence of RNAi (Table 1) [111]. Resistance against specific virus infections can readily occur by mutation of proviral genes encoding “viral sensitivity factors”, i.e., cellular factors that are required for efficient entry, replication and exit of a specific virus (Table 1). On the other hand, more broad antiviral mechanisms also exist, such as those involving non-specific RNA degradation (Table 1). In *Drosophila* populations, mutant flies can be identified that are resistant to viruses that naturally infect *Drosophila* but not to viruses with broad host range usually not encountered in nature [94]. Analysis of the mutants reveals antiviral defense mechanisms that are different from RNAi. Viruses also trigger apoptosis and phagocytosis of apoptotic virus-infected cells is recognized as a broad antiviral strategy [30,176].

How virus infection is recognized is still a major issue since only a limited number of PRRs recognizing viral PAMPs were identified such as the helicase domain of Dcr-2 and the Toll-7 receptor [26,147]. It is possible that virus infection is mainly detected in an indirect manner, for instance through the damage incurred by encoded virulence factors or excessive viral replication [154]. Release of cellular material such as proteases, heat-shock proteins and dsRNA subsequently may trigger the activation of classical immune response pathways such as Toll, Imd and Jak-STAT. Priming of these pathways may also function as prophylactic response against opportunistic bacterial or fungal infections and invasion of microbiota from the gut.

A major question concerns whether the “alternative” antiviral defense pathways can provide protection in the absence of RNAi. While not investigated systematically yet, it certainly seems possible given the vast spectrum of antiviral defense mechanisms that already have been described. In the case of *Wolbachia* infections, protection could be achieved in RNAi pathway mutants, indicating that RNAi is not necessarily essential to control viral infection [196]. Clearance of low level Nora virus infections and control of persistent infection could also occur in the absence of the RNAi machinery [202]. It is therefore interesting to investigate more systematically the relative contribution of RNAi in the antiviral response and whether RNAi efficiency is affected when other defense mechanisms predominate. While *Drosophila* can function as a useful model, this issue is particularly important as it can be considered as a limiting factor in RNAi efficiency and interfere with the successful application of RNAi products in the control of agricultural pests and vectors of diseases and the protection of beneficial insects from parasite diseases. A good example is the successful application of dsRNA in the syrup to increase the health of honeybee hives against dicistrovirus infections.

## Figures and Tables

**Table 1:** Overview of genome-wide or more targeted RNAi screens to identify cellular factors that affect virus infection in *Drosophila* tissue culture cells. Identification of proviral genes (encoding viral sensitivity factors) and antiviral genes (encoding viral resistance factors) is indicated. Only genes that have been validated in adult flies are included. Abbreviations: SINV, Sindbis virus; DXV, Drosophila X virus; RVFV, Rift valley fever virus; LACV, LaCrosse virus; DCV, Drosophila C virus; DENV, Dengue virus; KUN, Kunjin virus (strain of WNV); WNV, West Nile virus; IIV-6, Invertebrate iridiscent virus 6; FHV, Flock house virus; VSV, Vesicular stomatitis virus.

Virus Family	Virus	Cellular Process	Genes/Factors/Complexes	References
*Birnaviridae*	DXV	translation	Pelo/Hbs1 complex (proviral)	[135]
*Bunyaviridae*	RVFV	intracellular signaling	PKC98e (PKCε homolog) (proviral)	[121]
	RVFV	transcriptional pausing, induction of antiviral genes	P-TEFb (positive elongation factor) (antiviral)	[118]
	RVFV	induction of antiviral genes	FoxK transcription factor (antiviral)	[136]
	RVFV, LACV	cap-snatching of host mRNAs	-Decapping protein 2 (Dcp2) (antiviral) -Me31B, LSM7 (decapping activators) (antiviral)	[125]
	RVFV, LACV	cell cycle	-DNA replication factor A complex (antiviral) -CycA, cdc2, RnRs (proviral)	[125]
	RVFV	autophagy	-Atg5, Atg7, Atg18 (autophagy machinery) (antiviral) -Toll-7, Traf6 (signaling pathway) (antiviral)	[129]
	RVFV	chromatin remodeling	TIP60 histone acetyltransferase complex (antiviral)	[110]
	RVFV	nucleo-cytoplasmic shuttling	XPO1 (antiviral)	[110]
	RVFV, LACV	RNA sensor	Rm62 DEAD-box helicase (antiviral)	[114]
	RVFV	RNA degradation	-3′-to-5′ RNA exosome (dRrp6, dDis3, dRrp4, dRrp41) (antiviral) -exosome cofactor TRAMP complex (dMtr4, dZcchc7) (antiviral)	[115]
*Dicistroviridae*	DCV	translation	ribosomal proteins RpS6, RpL19 (proviral) ribosomal protein RACK1 (proviral) initiation factor eIF3j (proviral) Pelo/Hbs1 complex (proviral)	[126,128,135]
	DCV	endocytosis	Rab5 (proviral)	[134]
	DCV	vesicular transport	COPI coatamer (retrograde transport Golgi-ER) (proviral)	[134]
	DCV	fatty acid biosynthesis	SREBP, fatty acid synthase (proviral)	[134]
	DCV	RNAi (siRNA, miRNA)	Ars2, CBP20, CBP80 (antiviral)	[124]
	DCV	transcriptional pausing, induction of antiviral genes	P-TEFb (positive elongation factor) (antiviral)	[118]
	DCV	induction of antiviral genes	-Nup98 (nucleoporin with role in transcription) (antiviral) -FoxK transcription factor (antiviral) -B52 (virus-induced gene) (antiviral)	[136,137]
	DCV	intracellular signaling	ERK signaling pathway (dSos, dRas, dMek, dErk (rl), ksr, cnk) (antiviral)	[113]
	DCV	transmembrane signaling	-PVR receptor tyrosine kinase (antiviral) -Pvf2 ligand of PVR (antiviral)	[112]
	DCV	RNA degradation	Drosha (RNAi independent) (antiviral)	[122]
*Flaviviridae*	DENV	ER function	α-glucosidase (proviral)	[120]
	KUN, WNV DENV-2	ER function	signal peptidase complex (SPCS1, SPCS2) (proviral)	[116]
	DENV	vacuolar acidification	V-ATPase (proviral)	[120]
	DENV	unfolded protein response	DnaJ-1, CG3061 (proviral)	[120]
	DENV	endocytosis, vesicular transport	α-adaptin, cnir, lqf, synaptogyrin, Syx4, Syx13 (proviral)	[120]
	DENV	RNA metabolism	-RNA-binding proteins: bol, Unr, CG5205 (proviral) -3′–5′ exonuclease-like CG6744 (proviral)	[120]
	KUN	transcriptional pausing, induction of antiviral genes	P-TEFb (positive elongation factor) (antiviral)	[118]
	KUN, WNV, DENV	chromatin remodeling	TIP60 histone acetyltransferase complex (antiviral)	[110]
	KUN, WNV, DENV	nucleo-cytoplasmic shuttling	XPO1, aldolase (antiviral)	[110]
	KUN	induction of antiviral genes	-Nup98 (nucleoporin with role in transcription) (antiviral)	[137]
	DENV	transmembrane signaling	-PVR receptor tyrosine kinase (antiviral) -Pvf2 ligand of PVR (antiviral)	[111]
*Iridoviridae*	IIV-6	translation	Pelo/Hbs1 complex (proviral)	[135]
*Nodaviridae*	FHV	RNAi (siRNA, miRNA)	Ars2 (antiviral)	[124]
	FHV	glycerophospholipid metabolism	Ace, Cct1, Cct2, fu12, and san (proviral)	[130]
*Rhabdoviridae*	VSV	endocytosis	Rab5 (proviral)	[126]
	VSV	RNAi (siRNA, miRNA)	Ars2, CBP20, CBP80 (antiviral)	[124]
	VSV	autophagy	-Atg5, Atg7, Atg8a, Atg12, Atg18 (autophagy machinery) (antiviral) -Toll-7 (signaling pathway) (antiviral) -Akt, PTEN (signaling pathway) (proviral)	[26,101]
	VSV	transcriptional pausing, induction of antiviral genes	-NELF (negative elongation factor (antiviral) -P-TEFb (positive elongation factor) (antiviral)	[118]
	VSV	induction of antiviral genes	-Nup98 (nucleoporin with role in transcription) (antiviral) -FoxK transcription factor (antiviral) -B52 (virus-induced gene) (antiviral)	[136,137]
	VSV	intracellular signaling	ERK signaling pathway (dSos, dRas, dMek, dErk (rl), ksr, cnk) (antiviral)	[113]
	VSV	transmembrane signaling	-PVR receptor tyrosine kinase (antiviral) -Pvf2 ligand of PVR (antiviral)	[112]
	VSV	chromatin remodeling	TIP60 histone acetyltransferase complex (antiviral)	[110]
	VSV	nucleo-cytoplasmic shuttling	XPO1, aldolase (antiviral)	[110]
	VSV	RNA degradation	-3′-to-5′ RNA exosome (dRrp6, dDis3, dRrp4, dRrp41) (antiviral) -exosome cofactor TRAMP complex (dMtr4, dZcchc7) (antiviral)	[115]
*Togaviridae*	SINV	RNAi (siRNA, miRNA)	Ars2 (antiviral)	[124]
	SINV	cellular receptor for virus entry	dNRAMP (Mvl) (proviral)	[117]
	SINV	ER-associated protein degradation (ERAD) pathway, proteasome	dSEC61A, dVCP, dPSMD11 (proviral)	[119]
	SINV	transcriptional pausing induction of antiviral genes	-NELF (negative elongation factor (antiviral) -P-TEFb (positive elongation factor) (antiviral)	[118]
	SINV	induction of antiviral genes	-Nup98 (nucleoporin with role in transcription) (antiviral) -FoxK transcription factor (antiviral) -B52 (virus-induced gene) (antiviral)	[136,137]
	SINV	intracellular signaling	ERK signaling pathway (dSos, dRas, dMek, dErk (rl), ksr, cnk) (antiviral)	[113]
	SINV	transmembrane signaling	-PVR receptor tyrosine kinase (antiviral) -Pvf2 ligand of PVR (antiviral)	[112]
	SINV	RNA degradation	-3′-to-5′ RNA exosome (dRrp6, dDis3, dRrp4, dRrp41) (antiviral) -exosome cofactor TRAMP complex (dMtr4, dZcchc7) (antiviral)	[115]
	SINV	RNA degradation	Drosha (RNAi independent) (antiviral)	[122]

**Table 2:** Overview of transcriptome studies following viral infection in *Drosophila* adult flies or tissue culture cells. The method used for genome-wide transcriptome analysis is also indicated (microarray, RNAseq). Abbreviations: DCV, *Drosophila* C virus; DMelSV, Drosophila melanogaster Sigma virus; SINV, Sindbis virus; FHV, Flock house virus; VSV, Vesicular stomatitis virus; WSSV, white spot syndrome virus; CrPV, Cricket paralysis virus; SFV, Semliki forest virus; dpi, days post infection; hpi, hours post infection.

Virus	Tissue/Cells	Time point	Reference
DCV	whole flies thoracic injection (microarray)	1 and 2 dpi	[146,168]
DMelSV	whole flies (vertically transmitted) (microarray)	persistent infection	[200]
SINV	S2 cells (microarray)	5 dpi	[151]
FHV and RNA1 replicon	S2 cells (microarray)	12 and 24 hpi (FHV) 18 hpi (RNA1 replicon)	[130]
VSV	S2 cells (microarray)	4 hpi	[118]
DCV, WSSV (activated, inactivated)	S2 cells (microarray)	1 hpi	[181]
FHV, SINV	whole flies thoracic injection (microarray)	2 and 3 dpi (FHV) 4 and 8 dpi (SINV)	[34,168]
SINV replicon	whole flies RNA replicon (microarray)	constitutive RNA replication	[149]
SINV	Nup98-depleted DL1 cells (microarray)	2 hpi	[137]
DCV	whole flies, fat body thoracic injection (RNAseq)	24 hpi	[153]
DCV	S2 cells (microarray)	8 hpi, 24 hpi	[167]
DCV, CrPV	whole flies thoracic injection (RNAseq)	24 hpi	[167]
SFV	Jw18Wol (Wolbachia infected cell line) (RNAseq)	7 and 24 hpi	[52]
